# Current status of iridium-based complexes against lung cancer

**DOI:** 10.3389/fphar.2022.1025544

**Published:** 2022-09-23

**Authors:** Tongfu Yang, Minghui Zhu, Ming Jiang, Feng Yang, Zhenlei Zhang

**Affiliations:** ^1^ State Key Laboratory for Chemistry and Molecular Engineering of Medicinal Resources/Key Laboratory for Chemistry and Molecular Engineering of Medicinal Resources (Ministry of Education of China), Collaborative Innovation Center for Guangxi Ethnic Medicine, School of Chemistry and Pharmaceutical Sciences, Guangxi Normal University, Guilin, Guangxi, China; ^2^ School of food and biochemical engineering, Guangxi Science and Technology Normal University, Laibin, Guangxi, China

**Keywords:** iridium (III) complexes, anticancer, lung cancer, A549 cell, mitochondrial target

## Abstract

Lung cancer is one of the most common malignant tumors, with the highest mortality rate in the world, and its incidence is second only to breast cancer. It has posed a serious threat to human health. Cisplatin, a metal-based drug, is one of the most widely used chemotherapeutic agents for the treatment of various cancers. However, its clinical efficacy is seriously limited by numerous side effects and drug resistance. This has led to the exploration and development of other transition metal complexes for the treatment of malignant tumors. In recent years, iridium-based complexes have attracted extensive attention due to their potent anticancer activities, limited side effects, unique antitumor mechanisms, and rich optical properties, and are expected to be potential antitumor drugs. In this review, we summarize the recent progress of iridium complexes against lung cancer and introduce their anti-tumor mechanisms, including apoptosis, cycle arrest, inhibition of lung cancer cell migration, induction of immunogenic cell death, etc.

## Introduction

Lung cancer is a malignant tumor originating from the bronchial mucosa or glands of the lung, with high morbidity and mortality. According to the global cancer statistics in 2020, lung cancer is estimated to have 2.2 million new cancer cases and 1.8 million deaths, which is currently the second most commonly diagnosed cancer and the main cause of cancer death, accounting for about one 10th (11.4%) of the total diagnosed cancer and one fifth (18.0%) of the total cancer deaths (Sung et al., 2021). Lung cancer is the main cause of cancer incidence rate and mortality in men, while in women, the incidence is the third highest after breast and colorectal cancer, and the death rate is the second highest after breast cancer. In addition, it is estimated that 28.4 million new cancers will be found in the world in 2040, an increase of 47% over the corresponding 19.3 million cases in 2020 (Sung et al., 2021). Lung cancer can be mainly divided into non-small cell lung cancer (NSCLC) and small cell lung cancer (SCLC). Among all lung cancers, non-small cell lung cancer accounts for about 85%–88%, and small cell lung cancer accounts for about 12%–15% (Alexander et al., 2020). Non-small cell lung cancer can be divided into three categories according to its characteristics and treatment measures: adenocarcinoma, squamous cell carcinoma and large cell carcinoma (Grapatsas et al., 2017).

For localized lesions of lung cancer, surgery is the most common treatment, while chemotherapy is mainly used for patients with advanced spread or recurrence of lesions. Due to the poor prognosis of lung cancer, more than 90% of lung cancer needs chemotherapy (Zappa and Mousa, 2016). The effect of chemotherapy on SCLC is positive in both early and late stages, and even about 25% of early SCLC can be cured by chemotherapy (Mamdani et al., 2015). Chemotherapy is also the main method to treat NSCLC (Nagasaka and Gadgeel, 2018). At present, platinum antitumor drugs such as cisplatin, oxaliplatin and carboplatin combined with chemotherapy drugs such as paclitaxel, docetaxel and gemcitabine are mainly used to increase the survival rate of patients with NSCLC (Rossi and Di Maio, 2016). However, lung cancer patients showed different sensitivity to platinum chemotherapy drugs, and some patients relapsed within 6 months after treatment (Giaccone et al., 1998). Traditional platinum drugs take DNA as the main target, through the formation of adducts with DNA, prevent DNA replication and transcription, and promote cancer cell apoptosis (Ghosh, 2019). However, tumor cells are prone to develop resistance to platinum drugs, which also significantly limits its clinical application (Boulikas and Vougiouka, 2003). In addition, platinum drugs can cause serious side effects, such as nephrotoxicity, nausea, vomiting, bone marrow suppression, ototoxicity, etc., which further restrict their effective use (Oun et al., 2018). Therefore, in order to overcome the above shortcomings, researchers have been committed to developing a new generation of anticancer drugs to replace platinum drugs.

Considering the effectiveness of cisplatin and its derivatives, other transition metal complexes, such as ruthenium, gold, iridium, copper and iron-based complexes, have become a new generation of promising anticancer agents due to their potential anticancer properties and selective cytotoxicity (Ma et al., 2013; Gaur et al., 2018; Ma et al., 2019). However, unlike non platinum metal compounds such as ruthenium and gold, iridium complexes have received less attention in their potential antitumor activities in early studies. The main reason is that the chemical inertia of iridium itself makes its ligands difficult to dissociate, resulting in the inability of iridium complexes to interact with biological macromolecules (such as DNA, proteins, etc.) *in vivo*, so it is difficult to produce biological activities (Helm and Merbach, 1999; Lo and Zhang, 2012). Until the 21st century, researchers have successively found or synthesized iridium complexes with antitumor activity, so that metal iridium antitumor compounds can develop rapidly (Ruiz et al., 2012; Paitandi et al., 2014). In addition, compared with traditional platinum anti-tumor metal drugs, iridium complexes have advantages such as high stability, good water solubility, excellent phosphorescent properties, many coordination sites and easy transformation (Pracharova et al., 2018). They can form stable complexes with O^O, C^N and N^N bidentate ligands and are widely used in organic electroluminescence, biological fluorescence probes, chemical sensors and catalytic synthesis (Lucas et al., 2012). The study also found that iridium complexes have a new anti-cancer mechanism different from platinum drugs, and have high anti proliferative activity against cisplatin resistant cancer cells (Thomas et al., 2022). Therefore, iridium complexes may be potential candidates for the treatment of platinum resistant cancer.

## Cyclometalated iridium (III) complexes

**FIGURE 1:**
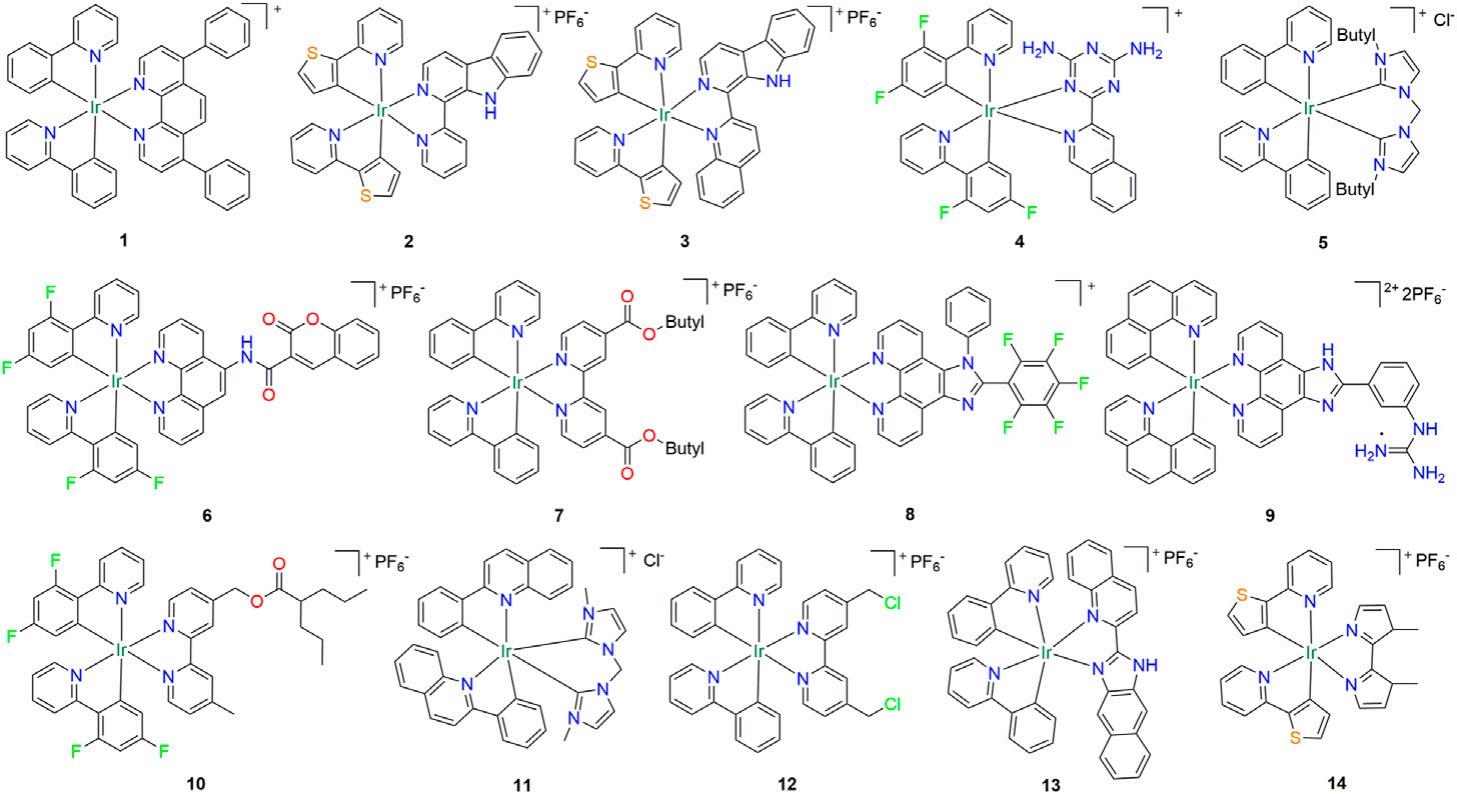
Structure of anti-lung cancer cyclometalated Iridium (III) compounds (**1–14**).

As early as the 1990s, cyclometalated iridium (III) complexes were reported because of their excellent luminescence properties (Mamo et al., 1997; Longato et al., 1999). In the next few years, cyclometalated iridium (III) complexes have become one of the most attractive phosphorescent heavy metal compounds in biological imaging and biosensing, due to their high quantum yield, large Stokes shift, long-lived luminescence, good photostability and cell permeability (Tamayo et al., 2003; Wilkinson et al., 2006; Monti et al., 2013). In 2013, Cao et al. Synthesized a series of cyclometalated iridium (III) complexes with 2,2′-bipyridine and 1,10-phenanthroline as ligands. These compounds showed good antitumor activity against HeLa cervical cancer cells and A549 lung cancer cells, and the inhibitory activity against A549 cells reached 2.0 μM. Due to its strong hydrophobicity, compound **1** (Figure 1) was found to effectively accumulate in the endoplasmic reticulum (ER), cause ER stress in cells, and further induce the intrinsic apoptotic pathway (Table 1) (Cao et al., 2013). After that, two cyclometalated iridium (III) complexes (**2** and **3**) (Figure 1), with 2-(2-thienyl)pyridine as an auxiliary ligand and β-carboline alkaloids as functional ligands, were synthesized by He and his co-workers (He et al., 2014). These two compounds not only showed strong anti-proliferative ability in a variety of cell lines, but also were equally effective in cisplatin resistant lung cancer cell lines (A549cisR cells) (Table 1). Further studies showed that compound **3** (Figure 1) could induce ROS mediated and caspase independent cell death through autophagy pathway in the absence of apoptosis, which indicated that its anticancer mechanism was different from that of cisplatin (Table 1). Xiong et al. Synthesized four cyclometalated iridium (III) complexes with 2,4-diamino1,3,5-triazine derivatives as the main ligands (Xiong et al., 2016). *In vitro* cytotoxicity experiments showed that the antitumor activity of compound **4** (Figure 1) was higher than that of cisplatin, and showed high selectivity between tumor cells and normal cells. This was because these compounds were taken up by A549cisR cells through an energy dependent pathway (Table 1).

**TABLE 1:** Cyclometalated Iridium (III) compounds as promising candidates against lung cancer.

No.	IC_50_ (µM)	Cell lines	Biology and mechanism	References
**1**	2.0 ± 0.1	A549	Cytotoxicity	Cao et al. (2013)
**2**	1.6 ± 0.2	A549	(1) Autophagic pathway	He et al. (2014)
	1.1 ± 0.1	A549cisR	(2) Caspase-independent cell death	
**3**	1.5 ± 0.1	A549	(3) Inhibition of mTOR signalling	
	0.86 ± 0.07	A549cisR	(4) Inhibition of CDK2	
**4**	2.77 ± 0.2	A549	(1) Producing ROS	Xiong et al. (2016)
	2.38 ± 0.4	A549cisR	(2) Acticating Caspase 9 and Caspase 3/7	
	5.64 ± 0.3	NCI-H460		
**5**	3.1 ± 0.4	A549	(1) Targeting mitochondria	Li et al. (2015)
	3.0 ± 0.2	A549cisR	(2) Phototoxicity	
**6**	0.62 ± 0.05	A549	(1) Mitochondrial damage	Ye et al. (2016)
	0.30 ± 0.02	A549cisR	(2) Photostability	
**7**	1.7 ± 0.1	A549	(1) Targeting mitochondria	Wang et al. (2016)
	2.1 ± 0.2	A549cisR	(2) Inducing autophagy and apoptosis	
**8**	0.8 ± 0.1	A549	(1) Targeting mitochondria	Ouyang et al. (2017)
	0.7 ± 0.2	A549cisR	(2) Inducing apoptosis	
**9**	13.65 ± 0.63	A549	(1) Targeting mitochondria	Song et al. (2017)
	13.63 ± 2.25	A549cisR	(2) Arresting cell cycle	
**10**	1.9 ± 0.2	A549	(1) Mitochondrial damage	Ye et al. (2017)
	0.79 ± 0.06	A549cisR	(2) Arresting cell cycle and inducing apoptosis	
**11**	1.7 ± 0.1	A549	(1) Targeting mitochondria	Li et al. (2017b)
	1.5 ± 0.2	A549cisR	(2) Photostability and inducing apoptosis	
**12**	0.4 ± 0.02	A549	(1) Targeting mitochondria	Cao et al. (2017)
	0.64 ± 0.04	A549cisR	(2) Causing cell ATP depletion	
**13**	0.31 ± 0.02 (Thomas et al.)	A549	(1) Photostability	Wang et al. (2017)
	0.72 ± 0.05 (Thomas et al.)	A549cisR	(2) Causing lysosomal damage	
**14**	11.2 ± 1.2	A549	(1) Inducing mitophagy	Chen et al. (2017)
	11.3 ± 1.1	A549cisR	(2) Causing cell ATP depletion	
**15**	3.6 ± 0.3	A549	Cytotoxicity	Yi et al. (2018)
**16**	1.4 ± 0.03	A549	Cytotoxicity	Tang et al. (2018)
**17**	0.93 ± 0.1	A549	(1) Targeting mitochondria	He et al. (2018a)
	1.0 ± 0.2	A549cisR	(2) Activating MAPK signaling pathway	
**18**	1.83 ± 0.20	A549	(1) Targeting mitochondria	Ouyang et al. (2018)
	2.46 ± 0.28	A549cisR	(2) Inducing cell necrosis	
**19**	3.4 ± 0.2	A549	Cytotoxicity	Zhang et al. (2019c)
**20**	1.2 ± 0.2	A549	Cytotoxicity	Du et al. (2019)
**21**	1.78 ± 0.30	A549	Cytotoxicity	Yang et al. (2019)
**22**	6.93 ± 0.44	NCI-H460	Cytotoxicity	Qin et al. (2018)
**23**	3.6 ± 0.4	A549	(1) Targeting lysosome	Chen et al. (2019)
	8.5 ± 0.3	A549cisR	(2) Inhibiting autophagic flux	
**24**	4.12 ± 0.14	A549	Targeting mitochondria	Hao et al. (2019)
**25**	0.25 ± 0.09	A549	Mitochondrial DNA damage and metabolism disturbance	Cao et al. (2019)
**26**	2.43 ± 0.13 (Hypoxia)	A549	(1) Targeting mitochondria	Li et al. (2019)
	1.29 ± 0.05 (Hypoxia)	A549cisR	(2) Inducing apoptosis	
**27**	0.5 ± 0.1 (Thomas et al.)	A549	(1) Photostability	Yuan et al. (2019)
	1.4 ± 0.5 (Thomas et al.)	A549cisR	(2) Targeting ER	
**28**	1.83 ± 0.1 (450 nm)	A549	(1) Photostability	Zhang et al. (2019a)
	0.52 ± 0.1 (450 nm + 405 nm)		(2) Photodynamic therapy	
	0.97 ± 0.3 (450 nm)	A549cisR	(3) Targeting mitochondria	
	0.45 ± 0.1 (450 nm + 405 nm)		(4) Inducing apoptosis	
**29**	1.1 ± 0.3 (HSA-Ir, light)	A549	(1) Targeting the nucleus	Zhang et al. (2019b)
	2.3 ± 0.2 (HSA-Ir, light)	A549cisR	(2) Photodynamic therapy	
	4.8 ± 0.2 (HSA-Ir, light)	A549 spheroid		
**30**	2.7 ± 0.2 (Ir-NH_2_)	A549	Cytotoxicity	Kuang et al. (2020)
**31**	0.69 ± 0.1	A549	(1) Targeting mitochondria	Li et al. (2020a)
	0.59 ± 0.06	A549cisR	(2) Inducing apoptosis	
**32**	1.5 ± 0.1	A549	(1) Photodynamic therapy	Li et al. (2020b)
	1.7 ± 0.1	A549cisR	(2) Targeting mitochondria	
**33**	>5 (405 nm)	A549	(1) Photodynamic therapy	Redrado et al. (2021)
	0.39 ± 0.09 (470 nm)		(2) Inducing apoptosis	
**34**	4.90 ± 0.21	A549	(1) Targeting ER	Wang et al. (2021)
	5.00 ± 0.31	A549cisR	(2) Inducing ICD	
**35**	2.27 ± 0.21	A549	(1) Targeting mitochondria	He et al. (2021)
	2.68 ± 0.27	A549cisR	(2) Inhibiting topoisomerase	
**36**	2.24 ± 0.04 (under ultrasound)	A549	Sonodynamic therapy	Xie et al. (2021)
**37**	5.4 (Thomas et al.)	A549	(1) Targeting lysosome	Huang et al. (2021)
	1.6 (Thomas et al.)	A549cisR	(2) Inducing apoptosis	
**38**	9.7 ± 0.15 (Lipo-Ir)	A549	Cytotoxicity	Gu et al. (2021)
**39**	4.9 ± 0.5	A549	Cytotoxicity	Zhou et al. (2021)
**40**	3.50 ± 0.17 (Dark)	A549	(1) Targeting lysosome	Redrado et al. (2022)
	0.26 ± 0.14 (Thomas et al.)		(2) Photodynamic therapy	
**41**	11.2 ± 1.1	A549	Cytotoxicity	Chen et al. (2022)
**42**	11.9	A549	Apoptosis	Ke et al. (2022)
**43**	41.42 ± 1.10 (Dark)	A549	Photodynamic therapy	Kuang et al. (2022)
	0.06 ± 1.80 (Thomas et al.)			
**44**	4.1 (Thomas et al.)	A549	(1) Targeting lysosome	Fan et al. (2022)
	2.5 (Thomas et al.)	A549cisR	(2) Inducing apoptosis	
**45**	1.8 ± 0.2	A549	(1) Targeting lysosome	Liu et al. (2022)
	4.9 ± 0.3	A549cisR	(2) Inducing apoptosis	
**46**	1.0 ± 0.1	A549	Cytotoxicity	Hao et al. (2022)
**47**	11.0 ± 0.4	A549	(1) Targeting mitochondria	Wu et al. (2022)
**48**	17.8 ± 0.3		(2) Inducing apoptosis	
**49**	0.53 ± 0.04 (Thomas et al.)	A549	(1) Targeting mitochondria	Xiong et al. (2022)
	0.83 ± 0.10 (Thomas et al.)	A549cisR		
**50**	1.90 ± 0.11 (Thomas et al.)	A549	(2) Phototoxicity	
	3.22 ± 0.15 (Thomas et al.)	A549cisR		
**51**	6.2 ± 0.2	A549	(1) Targeting mitochondria	Wang et al. (2022)
	3.3 ± 0.2	A549cisR	(2) Inhibiting metabolic	

The design of mitochondria targeted cytotoxic drugs represents a promising approach to selectively target tumors and overcome resistance to current anticancer therapies. Three cyclometalated iridium (III) complexes containing double N-heterocyclic carbene (NHC) ligands were developed by Li and his co-workers (Li et al., 2015). Interestingly, these iridium (III) complexes have strong fluorescence properties and mitochondrial targeting, which can rapidly and effectively penetrate into cancer cells and achieve therapeutic functions by simultaneously inducing and monitoring mitochondrial morphological changes. In addition, the cytotoxicity of compound **5** (Figure 1) to A549cisR cells under 365 nm light was 3488 times higher than that in the dark (Table 1). In 2016, three coumarin-appended phosphorescent cyclometalated iridium (III) complexes were explored as mitochondrial targeted anticancer agents (Ye et al., 2016). These compounds show abundant photophysical properties and can specifically target mitochondria. Among them, compound **6** (Figure 1) shows very high anti value-added activity against A549 cells and A549cisR cells. Mechanism studies have shown that this compound can exert its anticancer effect by initiating a series of events related to mitochondrial dysfunction (Table 1). In the same year, ten phosphorescent cyclometalated iridium (III) complexes containing 2,2′-bipyridine-4,4′-dicarboxylic acid and its diester derivatives as ligands are designed and synthesized by Wang and his co-worker (Wang et al., 2016). They found that the changes of ester substituents on the iridium (III) complex would affect the quantum yield, emission lifetime and cytotoxicity of iridium (III) complex. Among them, compound **7** (Figure 1) showed good anticancer activity against A549 cells and its cisplatin-resistant cells. Mechanism studies *in vitro* indicate that compound **7** undergoes hydrolysis of ester bonds, accumulates in mitochondria, and induces a series of cell-death related events mediated by mitochondria (Table 1). In addition, compound **7** can induce pro-death autophagy and apoptosis simultaneously. Through structural modification of compound **1**, Ouyang et al. synthesized six cyclometalated iridium (III) complexes with different fluorine atoms (Ouyang et al., 2017). These compounds also showed high inhibitory activity against A549 lung cancer cells and cisplatin-resistant cells. Compound **8** (Figure 1) with the largest number of fluorine atoms had the best activity, and its anti-tumor mechanism was similar to compound **7** (Table 1). Next, four cyclometalated iridium (III) complexes containing guanidine ligands were synthesized through structural modification of compound **8** (Song et al., 2017). Among them, compound **9** (Figure 1) has the best inhibitory activity on HeLa cells. Unfortunately, although its inhibitory activity against A549cisR is better than that of cisplatin, it is not as good as compound **8**. Compound **9** can not only selectively localize in mitochondria and induce cancer cell death through ROS-dependent pathway, but also block cell cycle in G0/G1 phase (Table 1).

Ye et al. designed and synthesized a valproic acid (VPA)-functionalized cyclometallized iridium (III) complex **10** (Figure 1) by conjugation of VPA with iridium (III) complexes *via* ester bond, which showed excellent two-photon properties and could be used for live-cell imaging (Ye et al., 2017). The ester bond in compound **10** was rapidly hydrolyzed by esterase and showed inhibition of histone deacetylase activity similar to VPA. Further studies of anticancer mechanisms revealed that compound **10** induced a series of events related to mitochondrial damage in cancer cells, including MMP depolarization, ROS production, cell cycle arrest, caspase activation, and apoptosis (Table 1). In 2017, four cyclometallized iridium (III) complexes containing N-heterocyclic carbene ligands were explored as mitochondrial targeted anticancer agents and photodynamic agents (Li Y. et al., 2017). These complexes show high ^1^O_2_ quantum yield in the presence of 450 nm LED light and can be used as effective visible light photosensitizers. The phototoxicity index of compound **11** (Figure 1) with the best activity to A549 and A549cisR were 500 and 789, respectively (Table 1). In the same year, the above research group obtained compound **12** (Figure 1) with better activity against A549 cells and cisplatin resistant cells by halogenating the ester group of compound **7** (Cao et al., 2017). The difference between the anti-tumor mechanism of compound **12** and previous compounds is that it cannot only cause mitochondrial damage, increase of ROS and induce caspase dependent apoptosis by targeting mitochondria, but also cause cell ATP depletion and mitochondrial respiratory inhibition (Table 1). Wang et al. designed and synthesized four phosphorous cyclometallized iridium (III) complexes containing benzimidazole moieties (Wang et al., 2017). Interestingly, compound **13** (Figure 1) has almost no dark cytotoxicity to A549 cells and A549cisR cells, but its phototoxicity index reaches 322 under 425 nm light. Compound **13** can effectively inhibit a variety of cancer processes, including cell migration, invasion, colony formation and angiogenesis, and can induce apoptosis through lysosomal damage and caspase-3/7 activation (Table 1). In addition, *in vitro* analysis of 70 kinases showed that maternal embryo leucine zipper kinase (MELK), PIK3CA and AMPK were potential molecular targets of the compound. Through structural modification of compound **2**, Chen and his colleagues synthesized phosphorous cyclometallized iridium (III) complex **14** (Figure 1), which can accumulate in mitochondria and induce mitotic phagocytosis through mitochondrial membrane potential depolarization, cell ATP depletion, mitochondrial metabolic state disturbance and oxidative stress (Table 1) (Chen et al., 2017).

**FIGURE 2:**
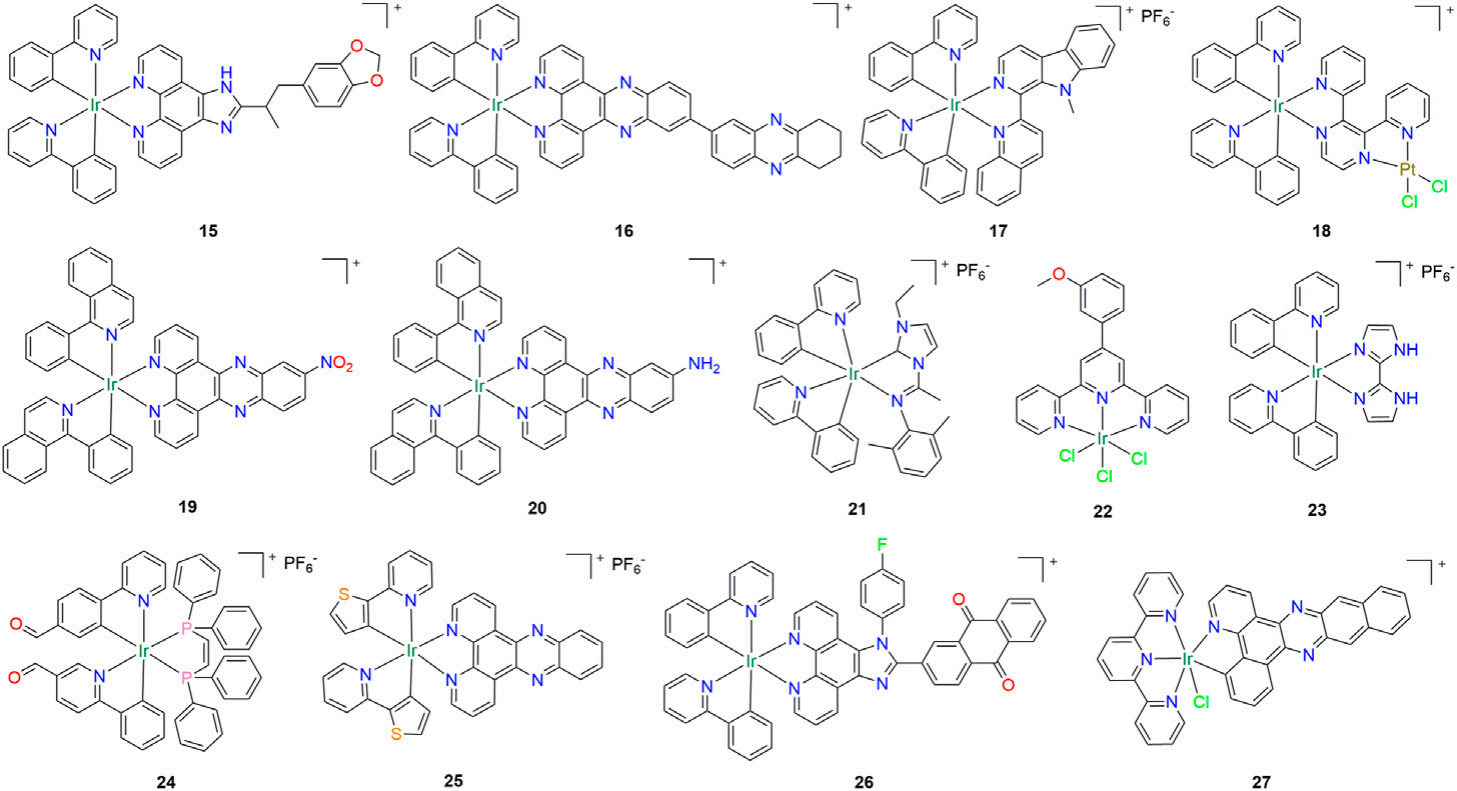
Structure of anti-lung cancer cyclometalated Iridium (III) compounds (**15–27**).

By modifying the structure of compound **9**, Liu and his colleagues successively synthesized two cyclometallic iridium (III) complexes **15** and **16** (Figure 2) (Tang et al., 2018; Yi et al., 2018). It was reported that both compounds **15** and **16** showed high inhibitory activity against A549 lung cancer cells, and both could induce apoptosis and induce autophagy to exert anti-tumor effects through the PI3K/mTOR signaling pathway (Table 1). In addition, the *in vivo* experimental results showed that the inhibitory rate of compound **15** on A549 xenograft tumor could reach 63.84%. Four cyclometallic iridium (III) complexes with good photophysical properties and anticancer activity were synthesized after structural modification of compound **3** by He et al. (He L. et al., 2018). Among them, compound **17** (Figure 2) can be quickly absorbed by A549 lung cancer cells, and it shows good inhibitory activity. The results of mechanism study showed that compound **17** could induce mitochondria derived cytoplasmic vacuolation by targeting mitochondria, and could also affect the ubiquitin proteasome system (UPS) and mitogen activated protein kinase (MAPK) signaling pathways (Table 1). *In vivo* studies showed that compound **17** could significantly inhibit tumor growth in mouse models, with an inhibition rate of 73%. In the same year, Ouyang et al. synthesized a hetero-binuclear Ir(III)–Pt (II) compound **18** (Figure 2) for the first time (Ouyang et al., 2018). *In vitro* cytotoxicity results showed that the compound **18** was effective against cisplatin-resistant tumor cells. Mechanistic experiments show that it can overcome cisplatin resistance by increasing cellular uptake, targeting mitochondria, and inducing cell necrosis (Table 1). Zhang and co-worker synthesized a cyclometallic iridium (III) complex **19** (Figure 2), which has the most significant antiproliferative effect on SGC-7901 human gastric adenocarcinoma cells and also shows good inhibitory activity on A549 lung cancer cells (Zhang W.-Y. et al., 2019). *In vitro* studies have shown that compound **19** can reduce the mitochondrial membrane potential of cancer cells and induce apoptosis by enhancing endogenous ROS and calcium levels (Table 1). At the same time, compound **19** is excellent in inhibiting tumor cell migration and inhibiting its G0/G1 phase growth. Compound **20** (Figure 2) was synthesized by reducing the nitro group in compound 19, and its inhibitory activity against A549 cells was increased by 3 times (Table 1) (Du et al., 2019).

A series of imine-N-heterocyclic carbene (C ˆ N) ligands with different substituents and their corresponding luminescent cyclometalated iridium (III) complexes were synthesized, in which compound **21** (Figure 2) entered A549 lung cancer cells *via* an energy-dependent pathway and targeted lysosomes (Yang et al., 2019). In this system, the larger steric hindrance of the ortho substituent in aniline and the longer length of alkyl substitution on the imidazole ring lead to the high anticancer activity of these cyclometalated iridium (III) complexes. Mechanistic studies showed that compound **21** exerted its anticancer effect mainly through cell cycle arrest, inducing apoptosis, increasing intracellular ROS levels and reducing mitochondrial membrane potential (Table 1). Qin et al. synthesized three iridium (III) complexes with terpyridine as ligand and showed selective cytotoxicity to tumor cell lines (Qin et al., 2018). Among them, compound **22** (Figure 2) has the best antitumor activity, which can not only trigger apoptosis of cancer cells through the mitochondrial dysfunction pathway, but also act as a telomerase inhibitor by directly targeting the c-myc promoter element (Table 1). After that, Chen et al. obtained a new cyclometalated iridium (III) complex **23** (Figure 2) through structural modification of compound **5**, in which 2-phenylpyridine was used as the ring metallization ligand and 2,2′-biimidazole was used as the auxiliary ligand (Chen et al., 2019). Due to the process of protonation and deprotonation of N-H group on 2,2′-biimidazole, compound **23** showed pH dependent phosphorescence and was able to specifically image lysosomes. Mechanism studies showed that compound **23** could induce caspase independent cell death through the elevation of ROS (Table 1). In addition, since it can alkalize lysosomes through anionic interference, it can also inhibit autophagic flux. Hao et al. designed and synthesized six cyclometallated iridium (III) complexes containing diphosphine ligands as mitochondria-targeted anticancer agents (Hao et al., 2019). They found that compound **24** (Figure 2) could impair mitochondrial energy metabolism and lead to a high production of mitochondrial reactive oxygen species (Table 1). Due to its viscosity-responsive phosphorescence lifetime, compound **24** was also able to monitor changes in mitochondrial viscosity in real time using two-photon phosphorescence lifetime imaging microscopy.

**FIGURE 3:**
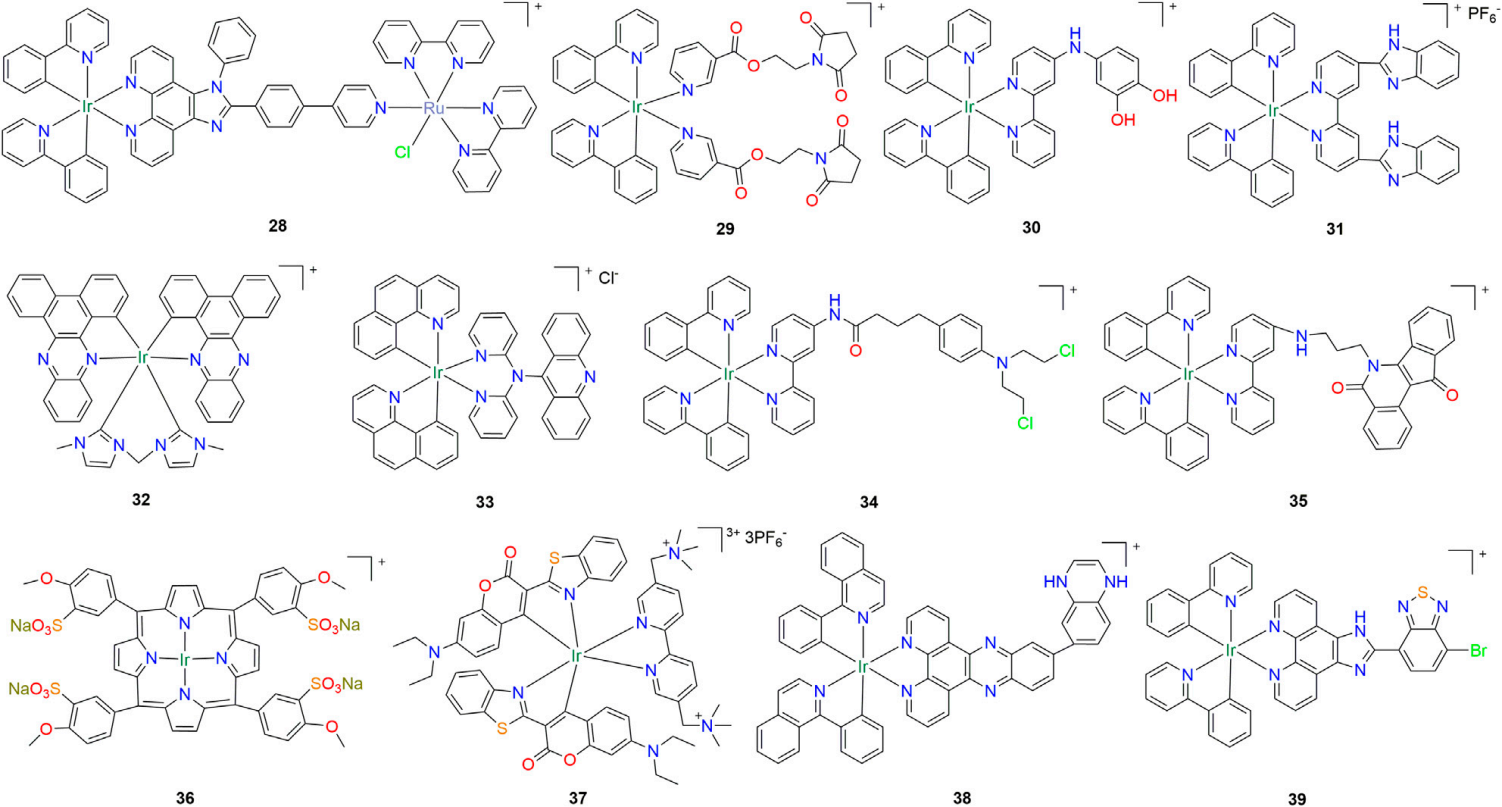
Structure of anti-lung cancer cyclometalated Iridium (III) compounds (**28–39**).

Mitochondrial DNA (mtDNA) is a potential target for cancer therapy. Cao et al. synthesized a series of cyclometallic iridium (III) complexes with dipyrido [3,2-a:2′,3′-c]phenazine (dppz) as ligand (Cao et al., 2019). Among them, compound **25** (Figure 2) has good anti-A549 lung cancer cell activity, which can tightly bind with DNA, insert mtDNA *in situ*, and induce mtDNA damage. The damaged mitochondria showed such phenomena as the decrease of mitochondrial membrane potential, the production of adenosine triphosphate, the interruption of mitochondrial energy and metabolic state, which led to protective mitochondrial phagocytosis, G0/G1 phase cell cycle arrest and apoptosis (Table 1). *In vivo* antitumor evaluation also showed that compound **25** could effectively inhibit the growth of tumor xenografts. In addition, in order to solve the problem of hypoxia limiting the efficacy of chemotherapy drugs in solid tumors, Li and co-workers synthesized a series of iridium (III) complexes with anthraquinone structure, in which compound **26** (Figure 2) can effectively respond to hypoxia by turning on yellow phosphorescence, and was successfully used to detect hypoxia in 3D multicellular tumor spheres (Li et al., 2019). The results of *in vitro* experiments showed that compound **26** showed good inhibitory activity against A549cisR cells under hypoxia (Table 1). Further studies showed that compound **26** preferentially accumulated in mitochondria of hypoxic tumor cells and induced apoptosis through mitochondrial dysfunction and caspase-3 activation. Yuan et al. synthesized a series of novel cyclometallic iridium (III) complexes with terpyridine and some bidentate ligands with increasing conjugation area, and found that they could specifically accumulate in the endoplasmic reticulum (ER) of A549 cells (Yuan et al., 2019). Among them, compound **27** (Figure 2) has good phototoxicity and can cause upregulation of CHOP and trigger ER stress-induced apoptosis in a short time after photodynamic therapy (405 nm), which highlights its potential as a photosensitizer candidate for ER localization photodynamic therapy (Table 1). Inspired by the structure and function of the compound **18**, Zhang et al. developed a dual-functional hetero-binuclear Ir-Ru compound **28** (Figure 3), which combines photoactivated chemotherapy and photodynamic therapy, with strong antitumor activity against cisplatin-resistant cancer cells (Zhang C. et al., 2019). Compound **28** enters cells by active transport and accumulates specifically in mitochondria. After light exposure, compound **28** could induce apoptosis through mitochondrial DNA damage and mitochondrial dysfunction (Table 1).

Zhang et al. synthesized cyclometal iridium (III) complex **29** (Figure 3) by modifying compound **7** and introducing a maleimide side chain (Zhang P. et al., 2019). Interestingly, compound **29** can react with human serum albumin (HSA) through the side chain to give HSA-Ir conjugates. Compared with the parent compound **29**, HSA-Ir exhibited significantly enhanced phosphorescence (Table 1), which could accumulate in the nuclei of live cancer cells and showed significant photocytotoxicity against A549 lung cancer cells and tumor spheroids, whereas even after light irradiation, Normal cells and normal cell spheroids remained nontoxic. This nuclear-targeted organoiridium albumin is a powerful candidate photosensitizer for antitumor photodynamic therapy. Kuang et al. reported an Fe(III) activated lysosome targeting Iridium (III) compound **30** (Figure 3) that can be used in the treatment of gastric cancer, and this compound also has a good inhibitory effect on A549 lung cancer cells (Kuang et al., 2020). It contains a meta aminocatechol group, which can selectively bind to and be oxidized by free Fe (III) in the cell. The subsequent oxidative rearrangement releases Fe II and hydrolyzes the amine bond under acidic conditions to form Ir-NH_2_ compounds. Ir-NH_2_ can migrate to mitochondria as a probe to reveal diagnostic information and induce cancer cell toxicity (Table 1). In the same year, Li et al. synthesized a phosphorus cyclometal iridium (III) complex **31** (Figure 3), which can effectively target mitochondria and has good antitumor activity against A549 lung cancer and cisplatin resistant cells (Table 1) (Li et al., 2020a). Another study by Che and co-worker reported that a near-infrared emitting iridium (III) N-heterocyclic carbene compound **32** (Figure 3) can be used as a mitochondrial targeting anticancer agent and photodynamic agent (Li et al., 2020b). This compound has greater cytotoxicity against A549 cancer cells than cisplatin and shows higher cytotoxicity in the presence of 450 and 630 nm LED light (Table 1). Compound **32** can increase intracellular ROS levels, reduce mitochondrial membrane potential, and induce early apoptosis to a certain extent. Under light, its tumor inhibition rate *in vivo* was 81%.

The next year, Redrado et al. obtained several novel luminescent and photosensitizing iridium (III) complexes through acridine modification, and studied their emission and biological activity against A549 cell line (Redrado et al., 2021). The results showed that the IC_50_ value of compound **33** (Figure 3) against A549 cells in the dark was 43.38 ± 0.14 µM, and the inhibitory activity was increased by 111 times when irradiated at 470 nm for 10 min (Table 1). Microscopic analysis showed that compound **33** could induce cytoplasmic vacuolization and typical apoptosis and necrosis. In the same year, Wang et al. designed and synthesized an iridium (III) complex **34** (Figure 3) that can target the ER and induce cell ER stress (Wang et al., 2021). It can induce multiple characteristics of immunogenic cell death (ICD) of non-small cell lung cancer cells (Table 1), namely, surface exposure of calreticulin, extracellular release of high mobility group protein box 1 (HMGB1) and ATP. This is the first report that iridium (III) complexes can cause ICD. He et al. also reported a new cyclometal iridium (III) complex **35** (Figure 3), which can effectively target mitochondria and inhibit mitochondrial topoisomerase (Table 1) (He et al., 2021). The next interesting report is that a new water-soluble iridium (III)-porphyrin sound sensitive compound **36** (Figure 3) was synthesized, which produced good killing effects on a variety of cancer cells (including A549 lung cancer cells) under ultrasound irradiation, and showed ultrasonic activation ability at a tissue depth of more than 10 cm (Table 1) (Xie et al., 2021). This study provides guidance for the development of metal sonosensitizer for the treatment of lung cancer. Huang et al. reported a water-soluble, luminescent, and photostable coumarin-functionalized cyclometallated iridium (III) complex **37** (Figure 3), which was primarily localized to the lysosomes and mitochondria of cancer cells (Huang et al., 2021). Compound **37** induced significant light-triggered cytotoxicity against a variety of cancer cells, while remaining nontoxic against several normal cell lines and under dark conditions (Table 1). Its principle of photocytotoxicity is mainly through ROS that changes the oxidative balance and mitochondrial membrane potential in cells, resulting in necrosis and apoptosis of cancer cells. Furthermore, compound **37** exhibits high *in vivo* biocompatibility and photocatalytic anticancer efficiency.

**FIGURE 4:**
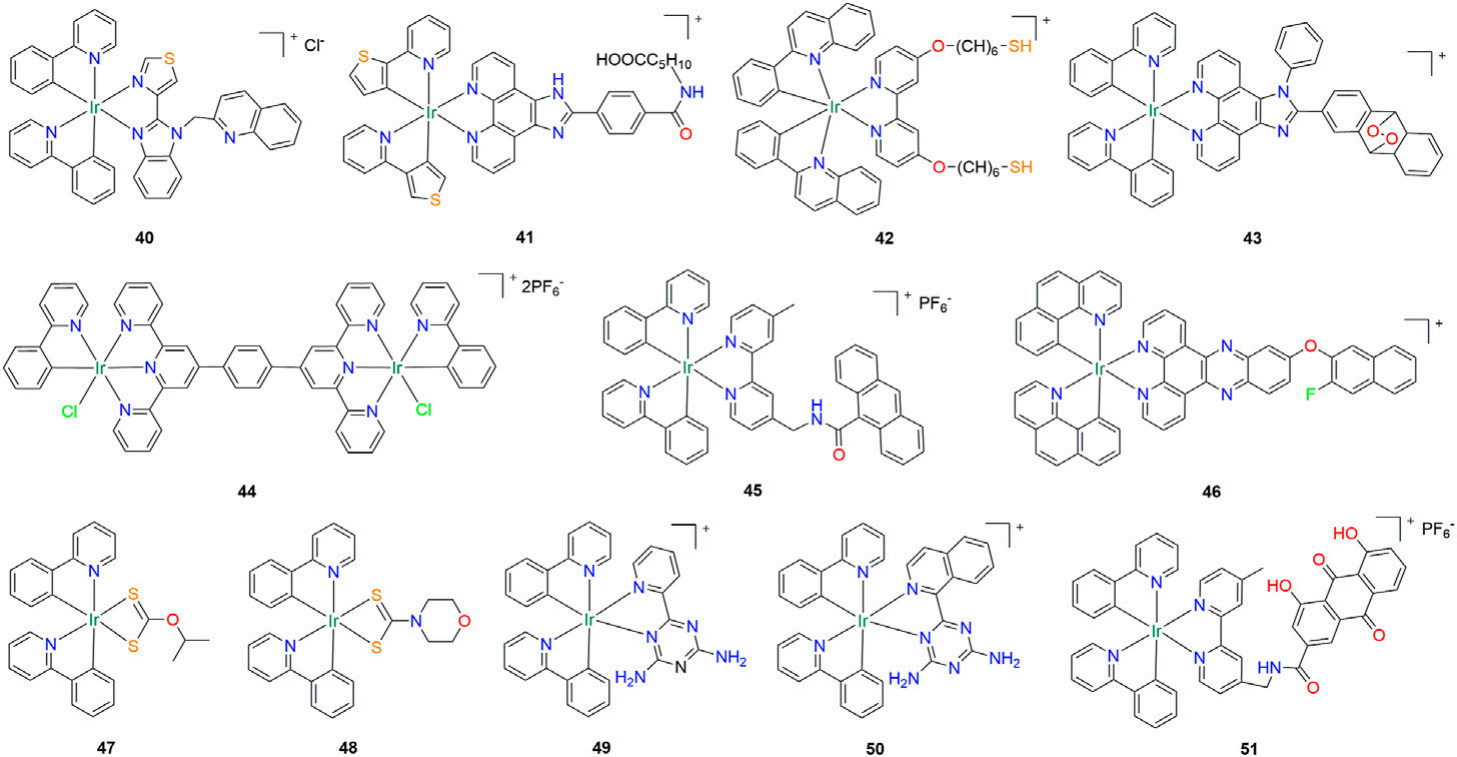
Structure of anti-lung cancer cyclometalated Iridium (III) compounds (**40–51**).

Liu’s group successively reported two cyclometallated iridium (III) complexes **38** and **39** (Figure 3) (Gu et al., 2021; Zhou et al., 2021). Among them, compound **38** exhibited a lower antitumor activity than compound **39**. However, after liposome loading, the IC_50_ value of compound **38** against A549 lung cancer cells reached 9.7 ± 0.15 µM. Another compound **39** can induce apoptosis of lung cancer cells by activating PI3K-Akt-mTOR and endoplasmic reticulum stress pathway, and inhibit migration of lung cancer cells by blocking mitotic process (Table 1). Recently, Redrado and co-workers synthesized a cyclometallated iridium (III) complex **40** (Figure 4) with biological activity and luminescent properties using benzimidazole derivatives as ligands, which can target lysosomes (Redrado et al., 2022). Under 470 nm light, its anti-proliferation activity against lung cancer A549 cells increased 15-fold to reach the IC_50_ value in nanomolar range (0.26 ± 0.14 µM) (Table 1). Drugs can be selectively delivered into tumor cells through carriers such as aptamers, antibodies, proteins and peptides, thereby reducing the toxic effects of drugs on normal cells. Chen et al. designed and synthesized a cyclometallic iridium (III) complex **41** (Figure 4), which can form a conjugate with the aptamer AS1411 (Chen et al., 2022). Due to the targeting ability of the aptamer, the conjugate can specifically bind to nucleolar proteins overexpressed on the surface of cancer cells and display strong fluorescent signals for tumor imaging and diagnosis (Table 1). Interestingly, Ke et al. reported a thiol-modified cyclometallated iridium (III) complex **42** (Figure 4), which can form iridium nanoparticles by self-assembly for targeted delivery to tumor sites (Ke et al., 2022). Further studies showed that nanoparticles can be decomposed into monomeric iridium compounds in lung cancer cells, and then selectively reduce glutathione levels, leading to mitochondrial oxidative stress (Table 1). Additionally, it was also found that nanoparticles can produce lipid peroxide, resulting in combined cell death in the cancer cells by apoptosis and ferroptosis. Next, the above research group reported a new mitochondrial-localized iridium (III) endoperoxide prodrug compound **43** (Figure 4), upon two-photon irradiation in NIR, synergistically releases a highly cytotoxic iridium (III) complex, singlet oxygen, and an alkoxy radical (Kuang et al., 2022). Compound **43** was found to be highly (photo-)toxic in hypoxic tumor cells and multicellular tumor spheroids in the nanomolar range (Table 1).

Fan et al. reported a novel photostable phosphorescent organometallic binuclear iridium (III) complex **44** (Figure 4), which is an excellent photosensitizer and efficient photocatalyst for NAD(P)H and amino acid oxidation with significantly higher photocytotoxicity at 525 nm than its mononuclear structure (Fan et al., 2022). In addition, compound **44** also possesses lysosome targeting and high biocompatibility, showing significantly high light-activated anticancer activity against A549 lung cancer cells and HepG2 tumor-bearing mouse models *in vitro* and *in vivo* (Table 1). Subsequently, Liu et al. synthesized iridium (III) complex **45** (Figure 4) with 9-anthracenecarboxylic acid as ligand, which also has lysosomal targeting ability and shows good cytotoxic activity against a variety of tumor cell lines, especially against A549 cells (Table 1) (Liu et al., 2022). Hao et al. also synthesized an iridium (III) complex **46** (Figure 4) with lysosomal targeting ability, which can induce cancer cell apoptosis by interfering with cell redox homeostasis and inhibiting PI3K/Akt/mTOR pathway (Table 1) (Hao et al., 2022). Iridium compounds **47** and **48** (Figure 4) synthesized by Wu et al. with dithioformic acid as ligand has the function of targeting mitochondria, which could induce apoptosis and inhibit migration of A549 cells (Table 1) (Wu et al., 2022). Similarly, Xiong et al. also developed iridium (III) complexes **49** and **50** (Figure 4) with mitochondrial targeting function using 1,3,5-triazine-2,4-diamine derivatives as ligands, which were effective against A549 and its cisplatin resistant cells exhibited strong phototoxicity (Xiong et al., 2022). Compound **49** can induce mitochondria-mediated cell death in A549cisR cells under 405 nm radiation, thereby overcoming drug resistance (Table 1). Based on compound **45**, Wang et al. synthesized a Rhein-modified cyclometallated Ir(III) compound **51** (Figure 4), which could precisely target mitochondria, induce severe mitochondrial damage and inhibit glycolytic bioenergetics, ultimately leading to death from ATP starvation (Table 1) (Wang et al., 2022). In addition, compound **51** can also regulate the cisplatin metabolic pathway in A549cisR cells, such as up regulating the inflow of CTR1 and down regulating the outflow of MRP2 transporter, thereby producing good anti proliferation performance against cisplatin resistant cancer cells.

## Organometallic half-sandwich iridium (III) complexes

In recent years, organoiridium (III) “half-sandwich” complexes have been proved to show significant anticancer activity. The first reported organic “half-sandwich” iridium (III) complex was discovered in 2011 by Sadler and co-worker (Liu et al., 2011). They synthesized a series of iridium (III) complexes with pentamethylcyclopentadienyl as ligand and confirmed that they have a good inhibitory effect on A2780 human ovarian cancer cells. This provides a very important basis for the development of anti-lung cancer Iridium compounds based on such structures.

**FIGURE 5:**
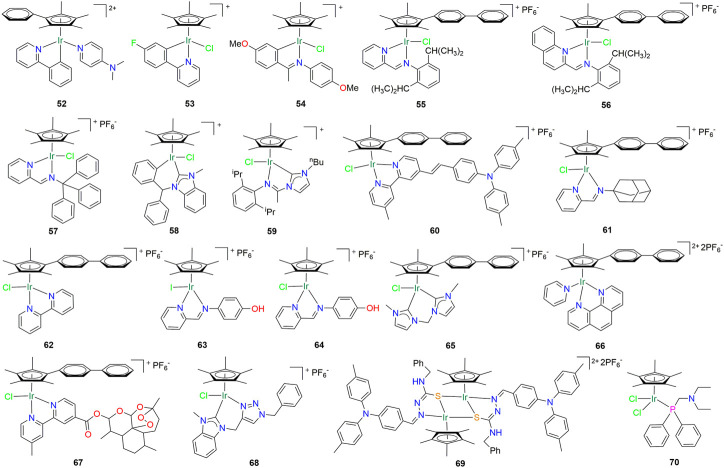
Structure of anti-lung cancer half-sandwich iridium (III) complexes (**52–70**).

Until 2014, Liu et al. synthesized a series of half-sandwich iridium (III) complexes with tetramethyl-(phenyl)cyclopentadienyl as ligands, among which compound **52** (Figure 5) showed a strong inhibitory effect on a variety of tumor cells (Liu et al., 2014). The IC_50_ of compound **52** against A549 cells was 0.62 ± 0.06 µM, which is 5-fold the antitumor activity of cisplatin (Table 2). In the next year, Millett and co-worker reported 15 half-sandwich Iridium (III) compounds (Millett et al., 2015). By modifying the functional groups at different positions of 2-phenylpyridine, it was found that the fluorine substituted compound **53** (Figure 5) had the best inhibitory activity on A549 lung cancer cells (Table 2). A series of “half sandwich” Schiff base IR (III) complexes were synthesized by Mou et al. (Mou et al., 2017). And their *in vitro* activity against leukemia K562 cell line was studied. They further found that compound **54** (Figure 5) had a good inhibitory effect on a variety of tumor cells, including A549 lung cancer cells, and induced apoptosis through the mitochondrial pathway (Table 2). Li et al. synthesized a series of novel half-sandwich iridium (III) complexes with neutral iminopyridyl Schiff base as ligands (Li J. et al., 2017). Among them, compounds **55** and **56** (Figure 5) showed high antitumor activity, and their activity against A549 and HeLa cells was 19 times and 6 times that of the clinical drug cisplatin, respectively (Table 2). These two compounds can be hydrolyzed in aqueous solution and do not interact with DNA, but have high binding affinity to albumin. In addition, compounds **55** and **56** could also significantly increase ROS levels in A549 cells, arrest cell cycle in G2/M phase, and induce apoptosis.

**TABLE 2:** Organoiridium (III) “half-sandwich” complexes as promising candidates against lung cancer.

No.	IC_50_ (µM)	Cell lines	Biology and mechanism	References
**52**	0.62 ± 0.06	A549	Cytotoxicity	Liu et al. (2014)
**53**	2.1 ± 0.3	A549	Cytotoxicity	Millett et al. (2015)
**54**	2.09	A549	Cytotoxicity	Mou et al. (2017)
**55**	1.4 ± 0.2	A549	(1) Arresting cell cycle	Li et al. (2017a)
**56**	1.1 ± 0.1		(2) Inducing apoptosis	
**57**	5.1 ± 0.3	A549	Cytotoxicity	Li et al. (2018)
**58**	3.9 ± 0.7	A549	Cytotoxicity	Han et al. (2018)
**59**	1.99 ± 0.1	A549	Cytotoxicity	Yang et al. (2018)
**60**	3.6 ± 0.5	A549	Cytotoxicity	He et al. (2018b)
**61**	2.7 ± 0.1	A549	Cytotoxicity	Kong et al. (2018)
**62**	14.7 ± 0.4	A549	Cytotoxicity	Zhang et al. (2018)
**63**	1.01 ± 0.08	A549	Cytotoxicity	Zhang et al. (2020b)
**64**	1.5 ± 0.3			
**65**	15	A549	Cytotoxicity	Zhang et al. (2020a)
**66**	4.4 ± 1.2	A549	Cytotoxicity	Liu et al. (2021)
**67**	1.82 ± 0.06	A549	Cytotoxicity	Chellan et al. (2021)
**68**	10 ± 1	NCI-H460	Cytotoxicity	Tong et al. (2021)
**69**	3.79 ± 1.15	A549	Cytotoxicity	Shao et al. (2021)
**70**	12 ± 3	A549	Cytotoxicity	Komarnicka et al. (2022)

Another work by Li et al., published in 2018, synthesized a half-sandwich iridium (III) complex **57** (Figure 5) using triphenyl-modified Schiff bases as ligands, which was mainly *via* energy-dependent active transport into cancer cells and accumulate in the nucleus (Li et al., 2018). In addition, compound **57** mainly induces apoptosis in the morning by increasing ROS levels in A549 cells and decreasing mitochondrial membrane potential (Table 2). Subsequently, Han et al. synthesized a series of N-heterocyclic carbene “half-sandwich” Ir(III) complexes, among which compound **58** (Figure 5) showed strong antitumor activity against A549 cells, which could target the lysosomes and mitochondria, and catalyze the conversion of the coenzyme NADH to NAD+, induce the production of reactive oxygen species, and can arrest the cell cycle in G1/S phase (Table 2) (Han et al., 2018). Furthermore, a series of novel and multifunctional imine cyclic carbene based “half sandwich” iridium (III) complexes were reported to have strong inhibitory activity against A549 cells (Yang et al., 2018). Among them, compound **59** (Figure 5) can reduce the mitochondrial membrane potential of A549 cancer cells, disrupt the G2/M phase cell cycle, and induce obvious apoptosis. Compound **59** enters A549 lung cancer cells mainly through an energy dependent pathway and is located in lysosomes (Table 2). In the same year, Liu’s research group successively reported three “half sandwich” Ir(III) complexes **60**, **61** and **62** (Figure 5) with cyclopentadiene dibenzene as ligand (He X. et al., 2018; Kong et al., 2018; Zhang et al., 2018). These three compounds have good inhibitory activity on A549 lung cancer cells, and can kill cancer cells by changing the level of intracellular reactive oxygen species, inducing apoptosis and blocking cell cycle (Table 2).

Zhang et al. found that the antiproliferative activity of the half-sandwich iodine-substituted cyclopentadienyl iridium (III) azopyridine compound **63** was greater than that of the chloro-substituted compound **64** (Figure 5) against cancer cells (Table 2), which was due to the fact that the iodine-substituted compound could intracellularly reacts with the abundant tripeptide glutathione to further activate it to generate cytotoxic free radicals (Zhang W. Y. et al., 2020). It has been reported that N-heterocyclic carbenes ligand modified half sandwich iridium (III) complex **65** (Figure 5) can enter lung cancer cells through an energy dependent pathway and target lysosomes to induce the release of cathepsin and other proteins (Zhang J. et al., 2020). These proteins regulate lysosomal and mitochondrial dysfunction, thereby promoting apoptosis (Table 2). At the same time, compound **65** can also block the cell cycle in G0/G1 phase. By replacing the ligand in compound **65** with phenanthroline, Liu et al. synthesized a series of new half-sandwich structure iridium (III) complex **66** (Figure 5), which had better inhibitory activity on A549 lung cancer cells than compound **65** (Table 2) (Liu et al., 2021). Furthermore, in contrast to complexes containing halide ion-leaving groups, pyridyl-based complexes do not show hydrolysis, but effectively cause lysosomal damage, leading to accumulation in the cytosol, inducing an increase in intracellular reactive oxygen species levels and apoptosis. Chellan et al. reacted dihydroartemisinin with 4-methyl-4′-carboxy-2,2′-bipyridine to generate new ester derivatives, and then synthesized several organometallic half-sandwich chloro Ir(III) complexes with it as ligand (Chellan et al., 2021). Compound **67** (Figure 5) has good inhibitory activity against A549 lung cancer cells (Table 2), and it also shows nanomolar antimalarial activity, which is superior to chloroquine and artemisinin.

Subsequently, a half sandwich Ir(III) compound **68** (Figure 5) with triazolyl substituted N-heterocyclic carbene as ligand was reported, which has good antitumor activity against NCI-H460 lung cancer cells (Table 2) (Tong et al., 2021). In the same year, Shao et al. developed four triphenylamine-modified fluorescent half-sandwich iridium (III) thiosemicarbazone (TSC) compounds, which exhibited fluorescence properties under 405 nm light (Shao et al., 2021). These complexes form unique dimer configurations due to the “enol” configuration of the TSC ligands. Among them, compound **69** (Figure 5) has the best inhibitory effect on A549 cells (Table 2). It can not only enter tumor cells in an energy dependent manner, accumulate in lysosomes, and cause damage to lysosomal integrity, but also block cell cycle and improve the level of reactive oxygen species in tissues, and lead to cell apoptosis. Recently, Komarnicka and co-workers synthesized two phosphine ligand-modified half-sandwich iridium (III) complexes, both of which showed strong inhibitory activity against A549 cells. Interestingly, compound **70** (Figure 5) induced cell cycle arrest in S phase at lower concentrations, but enhanced G0/G1 arrest at high doses (Table 2).

## Conclusion and prospects

Lung cancer is still one of the most vulnerable malignant tumors that seriously threaten human life. Although cisplatin and other platinum drugs are still the first choice for advanced chemotherapy of lung cancer, their toxicity, side effects and drug resistance limit their clinical use. In order to design and synthesize effective anticancer drugs, transition metal-based compounds have gradually developed into promising candidate drugs due to their cytotoxicity and ability to prevent drug resistance of tumor cells.

In recent years, iridium (III) complexes have been developed as potential anti-lung cancer drugs, promising to solve the toxic side effects and drug resistance of cisplatin. This paper reviews the recent progress of iridium (III) complexes, and discusses their biological activities and anti-lung cancer mechanisms. Currently, iridium (III) complexes that can effectively inhibit lung cancer are mainly divided into two categories, namely, cyclometallic iridium compounds and half-sandwich iridium compounds. Compared with half sandwich iridium compounds, cyclometallic iridium compounds have better optical properties and can inhibit tumors by exciting the dynamic behavior of light, which provides a basis for the development of Iridium based phototherapy reagents.

Most studies show that iridium (III) complexes have low toxicity to normal cells, are more easily taken up by tumor cells, and can effectively target lysosomes, mitochondria and endoplasmic reticulum of lung cancer cells. The anti-tumor mechanism of iridium (III) complexes is different from that of cisplatin. It mainly induces apoptosis or autophagy and cell cycle arrest by inducing ATP depleted mitochondrial damage, increased intracellular ROS level and endoplasmic reticulum stress, thus inhibiting cell proliferation, invasion and metastasis. Some iridium (III) complexes can also kill lung cancer cells by causing immunogenic cell death and inhibiting energy metabolism. In addition, in view of the inevitable low targeting and side effects of metal drugs, some new drug carriers, such as human serum albumin, have been used to improve the *in vivo* delivery efficiency, bioavailability and targeting, while greatly reducing the *in vivo* toxicity.

The existing research results clearly support that iridium (III) complexes can be used to develop effective chemotherapeutic drugs for human lung cancer, and they can provide guidance for the design of other metal drugs with higher efficiency and better clinical application potential.
